# Predicting Individuals at Risk of Diabetes Using Machine Learning

**DOI:** 10.7759/cureus.109761

**Published:** 2026-05-27

**Authors:** Brenda M Salas-Ramos, Irma Garcia-Calvillo, Jesus A Navarro-Acosta, Gloria Isela Mendoza-Frías

**Affiliations:** 1 Center of Interdisciplinary Studies and Research, Universidad Autonoma de Coahuila, Saltillo, MEX; 2 Center of Research in Applied Mathematics, Universidad Autonoma de Coahuila, Saltillo, MEX; 3 Department of General Surgery, Hospital Universitario de Saltillo, Saltillo, MEX

**Keywords:** class imbalance, clinical decision support, diabetes mellitus, early detection, machine learning, risk prediction

## Abstract

Diabetes mellitus (DM) is a serious global health problem due to the large number of people who suffer from it, the complications arising from it, and the significant economic impact associated with it. Early identification of people at risk is essential for implementing preventive strategies that reduce the burden on healthcare systems. In this context, machine learning (ML) techniques have emerged as promising tools to support the timely detection of diabetes and clinical decision-making.

The objective of the research was to develop and evaluate the performance of ML models to predict diabetes risk using clinical and sociodemographic data obtained at a primary care clinic of the Instituto Mexicano del Seguro Social (IMSS) in Saltillo, Coahuila, Mexico, the main healthcare system in Mexico. Data from 1,903 patients were analyzed, taking into account demographic and clinical variables and health habits. The database is not balanced; there are more healthy people than sick people. Supervised algorithms such as support vector machine (SVM), logistic regression (LR), random forest (RF), multilayer perceptron (MLP), and k-nearest neighbors (KNN) were implemented. In addition, to improve performance, ensemble techniques and class balancing methods, such as the Synthetic Minority Oversampling Technique (SMOTE), were applied. Performance was evaluated using metrics such as sensitivity and specificity, among others.

The results show that the models predict people without diabetes risk (class 0) with a high percentage, with sensitivities greater than 90% in several algorithms. In contrast, for detecting people at risk (class 1), the percentages are low, with sensitivities between 25% and 40% in the base models. The application of SMOTE and ensemble techniques, such as Extreme Gradient Boosting (XGBoost), increased sensitivity to 79%, although with an associated reduction in specificity (51.9%). These results show that, although ML models have remarkable potential to support the identification of diabetes risk, class imbalance remains a critical challenge.

Improving the sensitivity of these tools is essential to promote confirmatory studies, facilitate early treatment initiation, and reduce the onset of chronic complications. In addition, interpretable and clinically verifiable models can strengthen doctor-patient communication, encourage self-care, and promote the early adoption of preventive interventions aligned with international public health recommendations.

## Introduction

Diabetes mellitus (DM) is a serious global public health problem due to its increasing prevalence, multiple associated complications, and high economic impact. The International Diabetes Federation (IDF) reported in its Diabetes Atlas [1] that in 2024, approximately 3.4 million adults aged 20 to 79 died from the disease or its complications, including 2.4 million diagnosed cases and one million undiagnosed cases. In economic terms, the IDF reports that, in that same year, global health spending related to diabetes exceeded US$1 billion for the first time, highlighting the magnitude of the challenge that diabetes poses to health systems worldwide.

In general, there are three main types of diabetes: type 1, which is autoimmune in origin; type 2, which is associated with insulin resistance and lifestyle factors; and gestational diabetes, which occurs during pregnancy. Ninety percent of cases worldwide are type 2 diabetes and are associated with obesity, physical inactivity, and genetic factors [2].

The diagnosis of DM is confirmed by fasting plasma glucose tests, glycated hemoglobin (HbA1c) tests, and glucose tolerance tests, endorsed by the American Diabetes Association (ADA) and the World Health Organization (WHO). However, these tests are expensive and sometimes difficult to access in rural or low-income communities where diagnostic tests are not routinely available. Therefore, prevention and early diagnosis are key to reducing costs and complications from the disease [1, 2, 3].

Artificial intelligence (AI), particularly machine learning (ML) techniques, has become a valuable tool for predictive diagnosis and the early detection of chronic diseases such as diabetes [4]. In this context, several studies have explored the application of ML and deep learning techniques to improve risk prediction. For example, Ayoade et al. (2025) [5] conducted a comparative analysis of various ML and deep learning models, including support vector machine (SVM), random forest (RF), logistic regression (LR), and neural networks, to predict diabetes progression. Their study involved preprocessing clinical datasets from the Pima Indians, selecting relevant features, and evaluating model performance using metrics such as accuracy, precision, recall, and F1-score, demonstrating that ensemble and deep learning methods often outperform traditional approaches.

Similarly, Muhammad et al. (2020) [6] developed and evaluated supervised ML models for diabetes prediction using structured datasets collected through primary surveys at the Murtala Mohammed Specialist Hospital in Kano, Nigeria. Their work focused on implementing ML algorithms, emphasizing data preprocessing and model comparison. They assessed effectiveness using standard evaluation metrics and highlighted the importance of selecting appropriate algorithms to improve predictive performance.

In another study, Oladimeji et al. (2021) [7] proposed classification models aimed at early-stage diabetes prediction, incorporating feature selection techniques to identify the most relevant clinical attributes. Their methodology included dimensionality reduction to improve model efficiency and accuracy, demonstrating that feature selection significantly enhances classification performance in models such as logistic regression and decision trees.

Furthermore, Rana et al. (2024) [8] designed and implemented a web-based intelligent system for diabetes prediction using ML algorithms such as Extreme Gradient Boosting (XGBoost) and Naïve Bayes. Their approach integrated predictive models into an accessible digital platform, allowing users to input health-related parameters and obtain real-time risk assessments. 

Additional studies, such as that of Emi-Johnson and Nkrumah (2025) [9], have focused on identifying patients at higher risk of hospital readmission to support early interventions, using four ML models: LR, RF, XGBoost, and deep neural networks. Their findings demonstrated the potential of ensemble-based models to improve hospital discharge planning and reduce readmissions.

In general, these studies show that ML-based approaches can effectively identify patterns and relationships within large medical datasets, enabling diabetes risk prediction and supporting early clinical decision-making.

The literature described above demonstrates the application of ML techniques to diabetes prediction. Unlike these studies, this work uses real-world data from Instituto Mexicano del Seguro Social (IMSS), a clinic in the municipality of Saltillo, Coahuila, Mexico, which is inherently imbalanced, thereby more accurately reflecting real-world conditions and the challenges associated with clinical practice.

In this regard, the research addresses the problem of class imbalance in real-world contexts. The objective of this research is to contribute to the development of a predictive tool by applying ML algorithms to a database obtained from IMSS, a health clinic in the municipality of Saltillo, Coahuila, Mexico.

## Materials and methods

This research adopted an experimental and statistical approach based on a methodology of supervised learning techniques used in ML studies applied to health, biology, and engineering [10, 11].

This methodology was developed with a systematic and structured approach consisting of a series of consecutive stages [6, 12, 13] as follows: 1. Definition of labeled dataset; 2. Division of subsets: training and testing; 3. Model training: hyperparameter tuning with the training set; 4. K-fold cross-validation: repeated evaluation to avoid overfitting; 5. Final evaluation: calculation of performance metrics using test data; 6. Interpretation: analysis of variable importance and medical interpretation.

**Figure 1:**
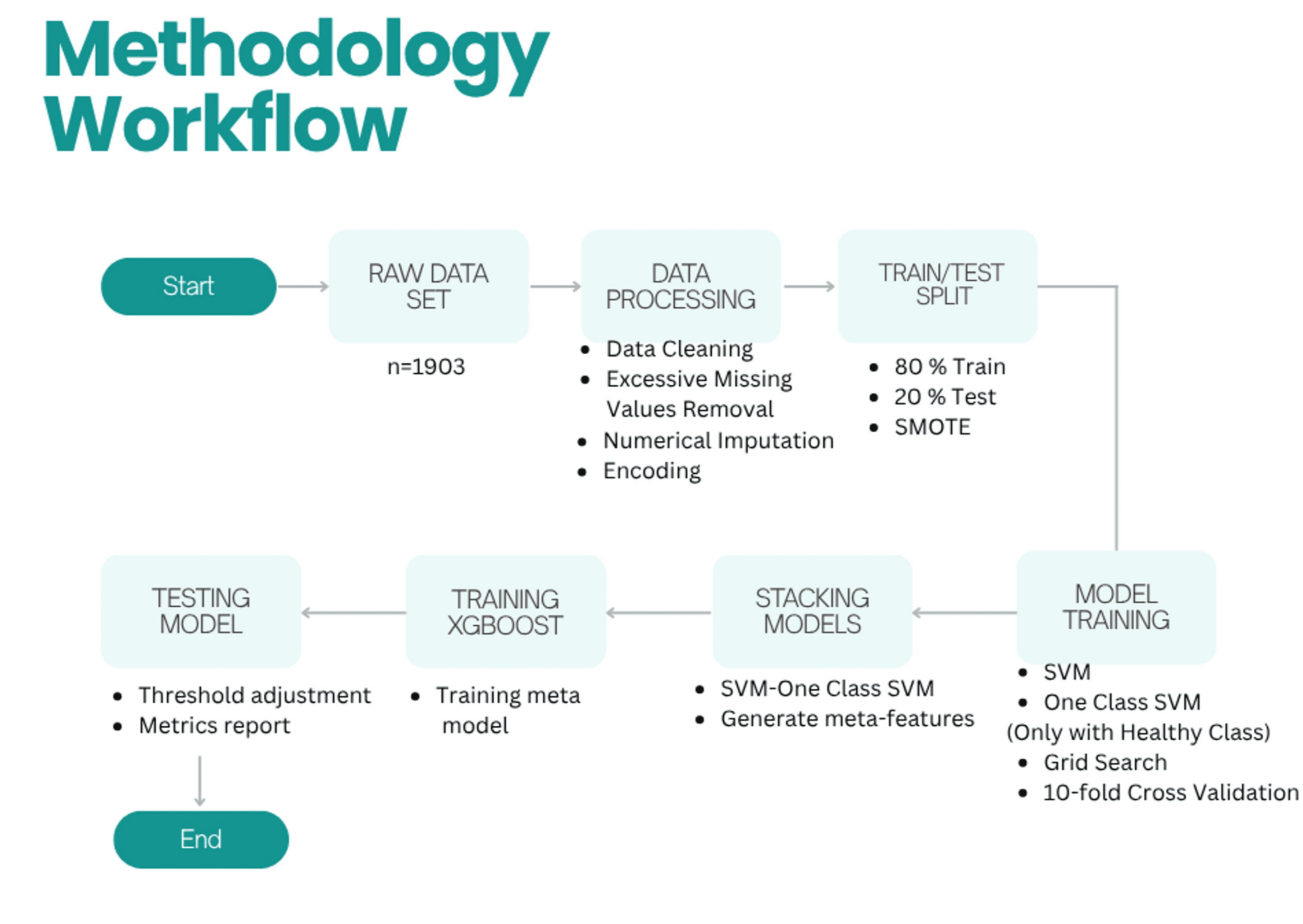
Workflow of the proposed methodology This diagram illustrates the process from data preprocessing to model evaluation. SMOTE: Synthetic Minority Oversampling Technique; SVM: support vector machine Figure created by the authors using Canva (Canva Pty Ltd., Sydney, Australia).

To provide a clear overview of the methodology applied to the best-performing technique for classifying individuals at risk or not at risk of diabetes, Figure 1 illustrates a flowchart summarizing each stage of the process, from the description of the initial dataset used and its preprocessing to model training, class imbalance handling, and final evaluation.

Database

The analysis was performed on the database generated by De la Rosa-De León et al. [14] from surveys of patients at a primary care clinic of the IMSS in Saltillo, Coahuila, Mexico, which included questions on demographic characteristics, medical history, and health habits relevant to diabetes. In the study, convenience sampling was used, considering individuals over 15 years of age who attended the consultation and were available during the data collection period.

The database contained information on 1,903 patients: 976 female and 915 male, aged between 15 and 84 years. Variables such as gender, age, body mass index, blood pressure, history of diabetes, glucose, soft drink consumption, triglycerides, cholesterol, diabetes-related symptoms, physical activity, city of origin, education, occupation, weight, and height were recorded. In this database, there was an imbalance, including 701 individuals with diabetes and 1,188 without diabetes, which showed a higher number of non-diabetic cases. Additionally, 14 records contained missing information or empty values, meaning that the diabetes status for those individuals was not available.

The diagnosis of diabetes was determined by clinical laboratory tests conducted prior to the medical consultation; patients with a fasting glucose level of 126 mg/dL or higher were diagnosed with diabetes. This represents the outcome variable. Data processing, analysis, and model development were performed using Python (version 3.12; Python Software Foundation, Wilmington, DE, USA). The following libraries were used: Pandas for data manipulation, NumPy for numerical computations, Scikit-learn for machine learning model implementation, Scikit-optimize (skopt) for hyperparameter optimization, Imbalanced-learn (imblearn) for handling class imbalance, and Seaborn for data visualization.

Preprocessing

The data underwent preprocessing that included statistical imputation. This imputation was performed using the k-nearest neighbors (KNNImputer) method for numerical variables and SimpleImputer (strategy=‘most_frequent’) for categorical variables. After imputation, the numerical variables were normalized using MinMaxScaler so that all variables were transformed to a comparable scale, thereby improving the performance of distance-based algorithms.

The dataset was split into training and test subsets using an 80/20 split via “train_test_split” with a fixed random state (random_state = 1234) to ensure reproducibility. To address class imbalance, the Synthetic Minority Oversampling Technique (SMOTE) was applied exclusively to the training set after the training/test split, thereby preventing data leakage and contamination of the test set with synthetic samples.

Training and ML techniques

The training process consisted of adjusting the hyperparameters of each classification algorithm, such as SVM, LR, RF, MLP, and KNN, by minimizing the error between predictions and actual labels. To avoid overfitting and evaluate the generalization capacity of the models, the k-fold cross-validation technique was implemented (cv=10), in which the training set is subdivided into partitions. The model is iteratively trained on subsets and validated on the remaining one until all the data is covered [15, 16]. This ensures a more stable performance estimate and reduces the variance related to a single data division [15, 16].

Ensembles

The application of ensemble learning or hybrid methods has become key to improving predictive power compared to individual methods. Applying ensemble learning compensates for errors or biases that occur in individual methods, thereby improving prediction rates.

The most commonly used techniques are bagging, boosting, and stacking. Bagging reduces the variance of the base estimator using samples called bootstraps. Boosting builds sequential models that correct previous errors, allowing for better performance. Some of its algorithms include Light Gradient Boosting (LightGBM) and XGBoost. Likewise, stacking improves prediction performance by combining models using a meta-model capable of learning advanced combination rules.

Therefore, we use these methods to optimize the performance of the classification model. [17, 18].

Metrics and analysis

After training the models, performance was evaluated using classification metrics such as accuracy, precision, sensitivity, specificity, and F1-score, which are important in clinical contexts to balance false positives and false negatives [19].

The utility of each metric and its corresponding formulas is described below.

Accuracy

Measures how well a model correctly classifies instances.

$Accuracy=\frac{(TP+TN)}{(TP+TN+FP+FN)}$

Sensitivity

Measures the proportion of actual positive cases that are correctly identified. It detects all diabetes cases.

$Sensitivity=\frac{TP}{(TP+FN)}$

Specificity

Measures the proportion of actual negative cases that are correctly identified. It correctly identifies healthy cases.

$Specificity=\frac{TN}{(TN+FP)}$

Precision

Measures how reliable the positive predictions of a model are.

$Precision:\frac{TP}{(TP+FP)}$

F1-Score

Measures the balance between precision and sensitivity of a model. It is used with imbalanced datasets because it is not biased by class distribution.

$F1Score=2\times \frac{(Precision)(Sensitivity)}{(Precision+Sensitivity)}$

In addition, other metrics are used, such as the Matthews correlation coefficient (MCC), which is used to evaluate binary classification models, especially in databases with imbalanced classes, such as the one in this study, where there are fewer patients with diabetes than healthy patients, and area under the receiver operating characteristic curve (AUC-ROC), which measures the model’s ability to distinguish between patients at risk for diabetes and those not at risk by comparing sensitivity and specificity metrics.

Finally, an analysis was performed to interpret the model, with the aim of identifying the contribution of clinical variables to predictions and making medical interpretations.

## Results

The performance of the ML models was evaluated using precision, recall, F1-score, and accuracy metrics. The analysis was performed by differentiating the behavior for class 0 (individuals without diabetes risk) and class 1 (individuals with diabetes risk), with the aim of identifying the ability of the algorithms to adequately discriminate between the two groups.

**Figure 2:**
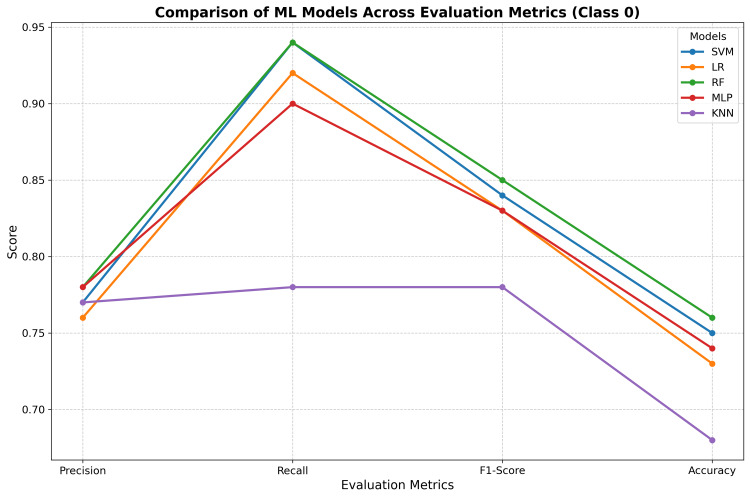
The graph represents the scores between 0 and 1 obtained for each metric for the machine learning (ML) techniques: support vector machine (SVM), logistic regression (LR), random forest (RF), multilayer perceptron (MLP), and k-nearest neighbors (KNN) in class 0, i.e., no risk of diabetes. This figure was created using Python (version 3.12; Python Software Foundation, Wilmington, DE, USA).

In class 0, corresponding to individuals without risk, the models showed favorable performance. Precision remained between 75% and 80% for all techniques evaluated, while recall exceeded 90% in most algorithms, with the exception of KNN. The F1-Score recorded values above 80%, and overall accuracy was around 75%, as shown in Figure 2.

Among the evaluated models, the best performance was achieved by the model corresponding to Case 8: stacking SVM + one-class SVM + XGBoost+adjusted threshold, which obtained the highest F1-Score (51.6%) and sensitivity (79%). These results indicate a superior ability to correctly identify individuals at risk of diabetes, which is crucial in clinical applications. Although this model presented lower specificity, resulting in a higher number of false positives, it is preferable in medical contexts where missing positive cases may have serious consequences.

**Figure 3:**
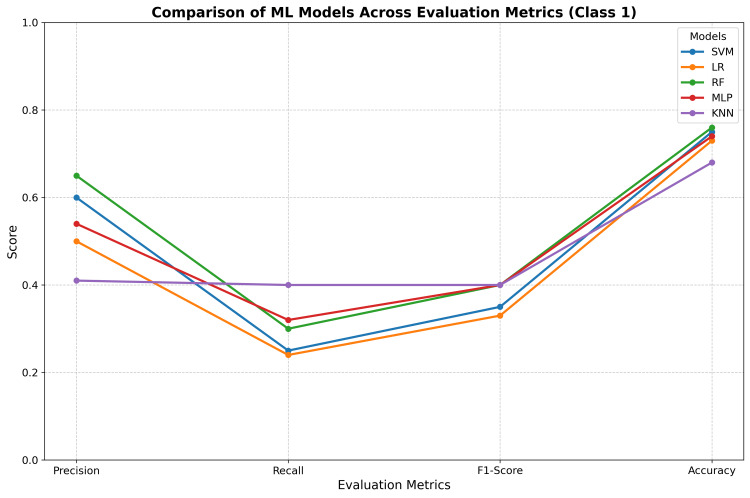
The graph represents the scores between 0 and 1 obtained for each metric for the machine learning (ML) techniques: support vector machine (SVM), logistic regression (LR), random forest (RF), multilayer perceptron (MLP), and k-nearest neighbors (KNN) in class 1, i.e., with diabetes risk. This figure was created using Python (version 3.12; Python Software Foundation, Wilmington, DE, USA).

In contrast, performance for class 1 (people at risk of diabetes) was considerably lower. Precision ranged from 40% to 60%, while recall ranged from 25% to 35%. The F1-Score ranged from 30% to 40%, while overall accuracy remained above 70%. These results are presented in Figure 3.

Next, the performance of ensemble techniques was analyzed using metrics derived from the confusion matrix, including sensitivity, specificity, precision, F1-Score, and accuracy. Table 1 summarizes the values obtained for each ML technique evaluated.

**Table 1:** Comparative table of confusion matrix results with ensembles The table shows the true negatives (TN), false positives (FP), false negatives (FN), and true positives (TP) produced by the confusion matrix and the accuracy, sensitivity, specificity, precision, and F1-Score metrics for each machine learning technique mentioned. SVM: support vector machine; SMOTE: Synthetic Minority Oversampling Technique; LightGBM: Light Gradient Boosting; XGBoost: Extreme Gradient Boosting

TECHNIQUE	tN	FP	FN	TP	Accuracy	Sensitivity	Specificity	Precision	F1-Score
One-class SVM normal (1)	234	30	75	25	71.2	25	88.6	45.5	32.3
One-class SVM optimized (2)	261	3	97	3	72.5	3	98.9	50	5.7
One-class SVM grid (3)	196	68	61	37	64.4	37.8	74.2	35.2	36.5
SVM with SMOTE (4)	176	88	38	62	65.4	62	66.7	41.3	49.6
SVM with SMOTE + OPT BAY (5)	215	49	67	33	68.1	33	81.4	40.2	36.3
Stacking SVM + one-class + LightGBM (6)	136	128	27	73	57.4	73	51.5	36.3	48.5
Stacking SVM + one-class + LR + adjusted threshold (7)	163	101	32	68	63.5	68	61.7	40.2	50.5
Stacking SVM + one-class + XGBoost + adjusted threshold (8)	137	127	21	79	59.3	79	51.9	38.3	51.6
Stacking SVM with SMOTE + one-class + XGBoost (9)	207	57	50	50	70.6	50	78.4	46.7	48.3

In general terms, heterogeneous behavior was observed among the models. Methods based on SVM in their basic configuration showed moderate accuracy but with limited sensitivity, particularly in one-class SVM optimized, where a value of 3% was recorded, indicating a very reduced capacity to correctly identify individuals at risk. On the other hand, applying oversampling techniques such as SMOTE substantially improved sensitivity (up to 62%), although with a corresponding decrease in specificity (66.7%). When SMOTE was combined with Bayesian optimization, sensitivity improved slightly (33%), although without exceeding the values achieved with simple SMOTE.

In relation to stacking models, the results show notable improvements. The combination of SVM with one-class and LightGBM (SVM + one-class + LightGBM) achieved a sensitivity of 73%, which represents a considerable improvement over individual models, although at the expense of specificity (51.5%). Likewise, threshold adjustment strategies in stacking models with LR and XGBoost showed a more favorable balance in positive class identification, achieving sensitivities of 68% and 79%, respectively. Finally, the hybrid stacking SVM with SMOTE, one-class and XGBoost methods without additional adjustment showed a balance between sensitivity (50%) and specificity (78.4%).

**Figure 4:**
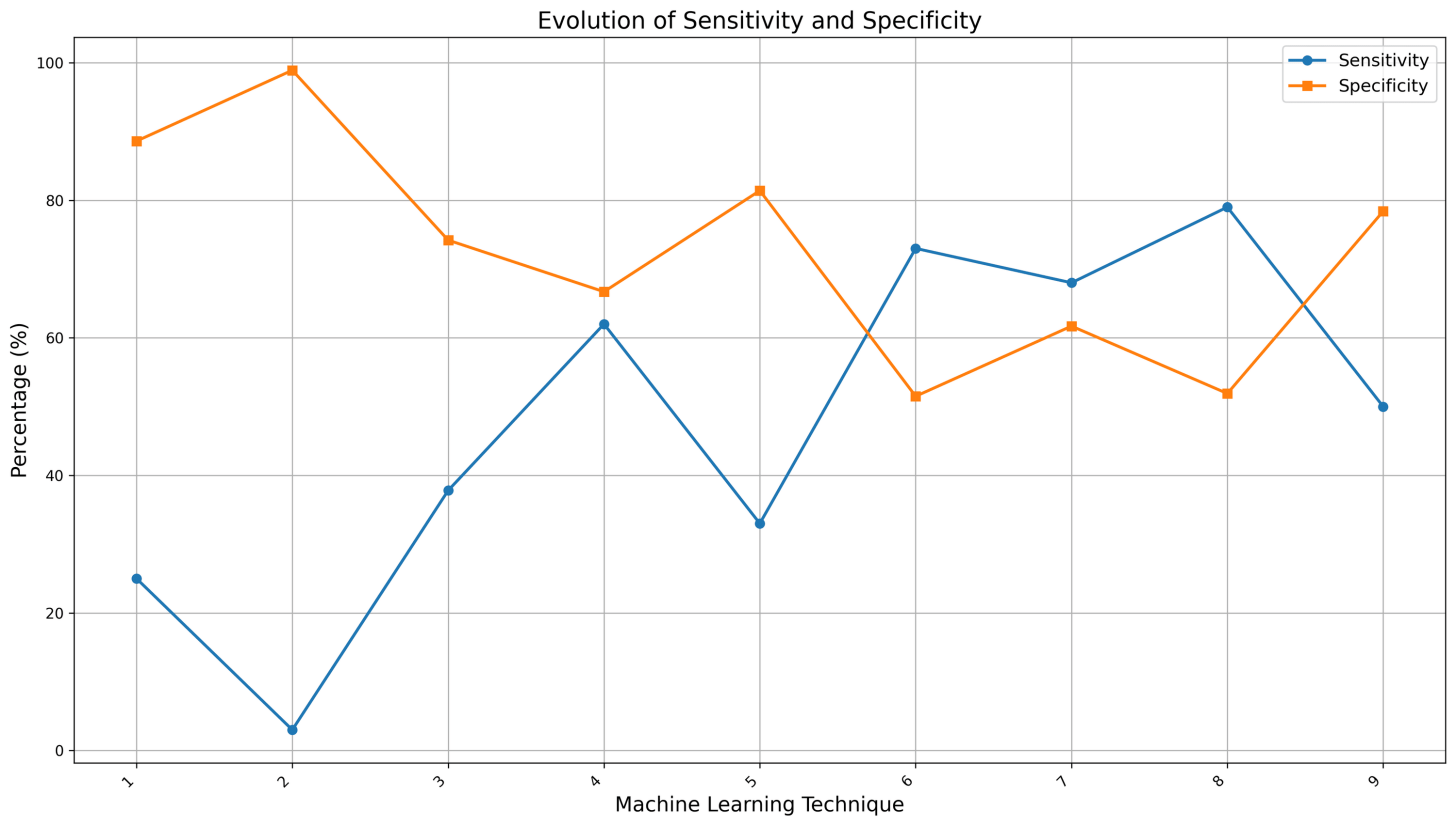
The graph represents the percentages obtained from the sensitivity and specificity metrics for the machine learning (ML) techniques: (1) one-class support vector machine (SVM) normal, (2) one-class SVM optimized, (3) one-class SVM grid, (4) SVM with Synthetic Minority Oversampling Technique (SMOTE), (5) SVM with SMOTE and Bayesian optimization, (6) stacking SVM + one-class SVM + Light Gradient Boosting (LightGBM), (7) stacking + logistic regression (LR) with adjusted threshold, (8) stacking SVM + one-class SVM + Extreme Gradient Boosting (XGBoost) with adjusted threshold, and (9) stacking SVM with SMOTE + one-class SVM + XGBoost. This figure was created using Python (version 3.12; Python Software Foundation, Wilmington, DE, USA).

Additionally, Figure 4 illustrates the evolution of sensitivity and specificity across the different techniques. Only these two metrics are included in this graph, given that they allow us to clearly visualize the difference between the detection of patients at risk (sensitivity) and the correct classification of individuals without risk (specificity). This visualization shows the pattern observed in the table: the initial models have high specificity at the expense of limited sensitivity, while the techniques based on stacking and threshold adjustment show the opposite trend, prioritizing the detection of class 1 but with a reduction in specificity.

Among the evaluated models, the best performance was achieved by the model corresponding to Case 8: stacking SVM + one-class SVM + XGBoost + adjusted threshold, which obtained the highest F1-Score (51.6%) and sensitivity (79%). These results indicate a superior ability to correctly identify individuals at risk of diabetes, which is crucial in clinical applications. Although this model presented lower specificity, resulting in a higher number of false positives, it is preferable in medical contexts where missing positive cases may have serious consequences.

**Figure 5:**
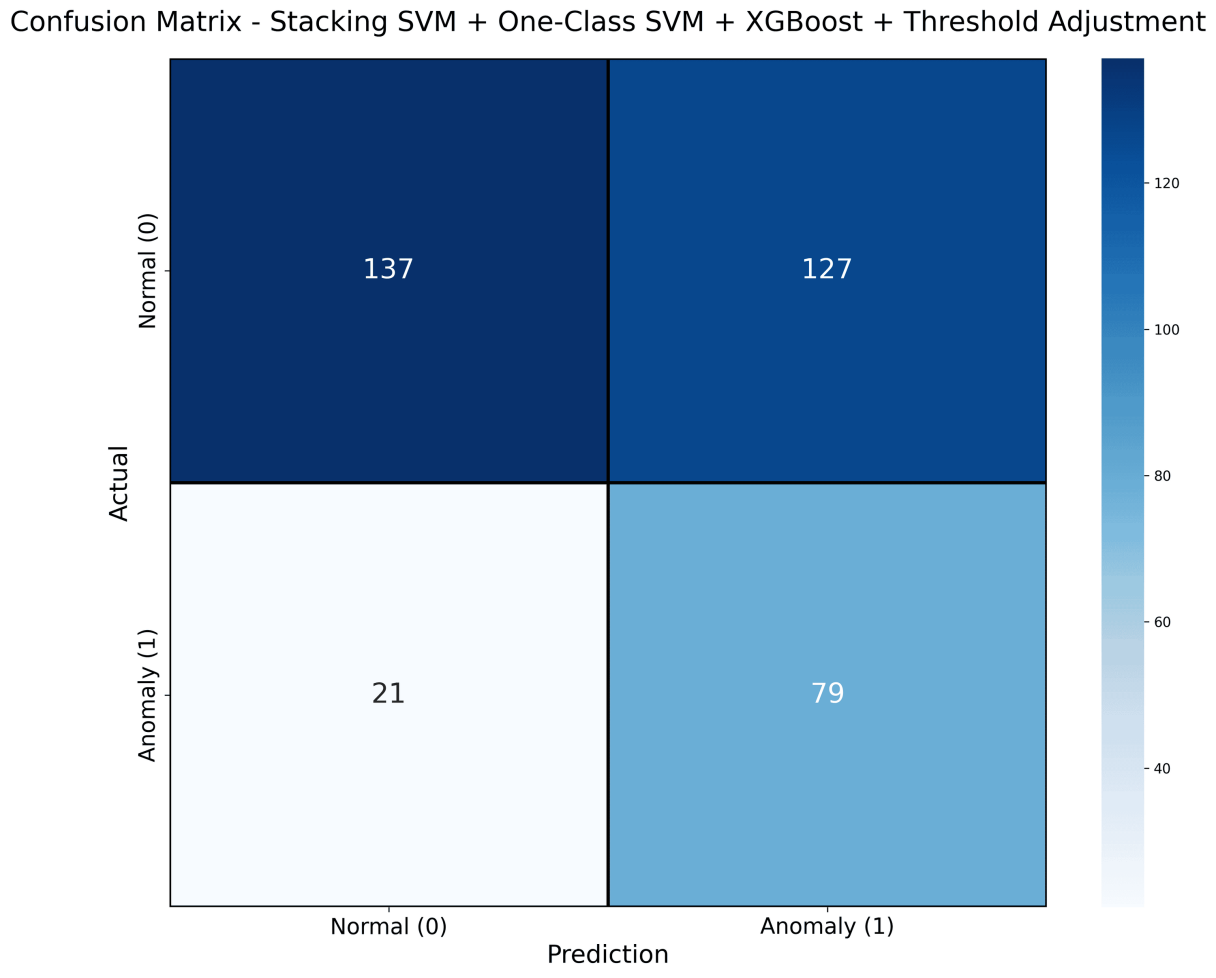
The figure shows a confusion matrix for a classification model combining stacking support vector machine (SVM), one-class support vector machine (SVM), and Extreme Gradient Boosting (XGBoost) with threshold adjustment. The matrix compares the predicted labels (x-axis) against the true labels (y-axis) for a binary classification task, where normal (0) represents the absence of diabetes and anomaly (1) represents the presence of diabetes. True negatives (TN): 137 cases correctly predicted as normal; false positives (FP): 127 normal cases incorrectly predicted as anomalies; false negatives (FN): 21 anomaly cases incorrectly predicted as normal; true positives (TP): 79 cases correctly predicted as anomalies. This figure was created using Python (version 3.12; Python Software Foundation, Wilmington, DE, USA).

Figure 5 shows the results of the confusion matrix for the best-performing method (stacking + XGBoost + adjusted threshold).

The color intensity reflects the number of samples in each cell, with darker blue representing higher counts. This visualization highlights that the model performs better at identifying normal cases than anomalous ones, as indicated by the higher number of true negatives compared to true positives.

Below are the results of the MCC and AUC-ROC metrics for the best-performing algorithm. The AUC-ROC value of 0.6795 indicates that the model is capable of distinguishing between patients at risk of developing diabetes and those who are not. This means that the clinical variables included in the database contain relevant information that the algorithm was able to partially utilize. However, the performance is not yet high enough to be considered a complete clinical model. An AUC close to 0.68 suggests that significant classification errors still exist, including both false positives and false negatives, consistent with the results of the metrics obtained previously.

Furthermore, the MCC value of 0.2762 reinforces this interpretation, as it shows a positive but relatively low correlation between the model’s predictions and the actual classification of patients. This may be due to the nature of the data or class imbalance.

Despite this, the confidence interval (0.6129, 0.7376) demonstrates that the model has a predictive ability that is statistically better than chance, which is important in preliminary medical applications.

To determine the contribution of each variable to the predictive performance of the models, a permutation feature importance analysis was used. This allowed us to identify the variables with the greatest influence on diabetes risk classification within the SVM model, the one-class SVM model, and the ensemble metamodel.

The variable importance analysis revealed that the metamodel relied more heavily on the probabilistic output of the SVM classifier (0.6187) than on the predictions generated by the one-class SVM model (0.3813), suggesting that the SVM made a more significant contribution to the ensemble’s final decision-making process.

The permutation importance analysis showed that, for the SVM model, the most influential variables were blurred vision, family history of diabetes, and age. Additional variables such as physical activity, male gender, and recent weight loss also showed positive contributions to predictive performance. These findings are clinically consistent with known risk factors and common symptoms associated with impaired glucose metabolism.

For the one-class SVM model, the most relevant variables included age, factors related to blood pressure and stress, weight loss, and symptoms such as polydipsia. Although the magnitudes of contribution were smaller compared to the conventional SVM model, these variables still demonstrated relevance for identifying anomalous patterns or high-risk patients.

Some variables showed negative values in permutation importance, indicating that their contribution to predictive performance was limited or potentially redundant within the current dataset. This behavior may be related to correlations among variables, sampling characteristics, or the exploratory nature of the study.

## Discussion

The first piece of evidence found is the difficulty ML algorithms have in detecting people at risk of diabetes (class 1). This situation is consistent with having an unbalanced database, which often affects the detection of positive cases [20, 21].

Not only does class imbalance affect the low performance reported in class 1, but it is also associated with reduced sensitivity, indicating that a considerable number of subjects at risk were not classified correctly, which may limit early preventive intervention. Therefore, several studies support that improving sensitivity over overall accuracy is extremely important in order to identify as many at-risk patients as possible [22, 23, 24].

On the contrary, class 0 (no risk) benefits from having a greater number of healthy people in the database, according to reports such as He et al. [25]. Techniques such as SMOTE, decision threshold adjustments, or differential cost strategies have been shown to improve performance in similar scenarios [21, 26].

In general, the results obtained determine the need to apply hybrid methods and/or class balancing strategies to improve the detection of patients at risk of diabetes. In addition to this, it is suggested that additional or clinical variables be added and broader data sets be used to improve the generalization of the model, in line with existing recommendations in the literature [3, 24].

The sensitivity reported by the models is a key point in the development of predictive tools applied to health, especially when used for population screening purposes. A technique with high sensitivity allows for the adequate identification of most individuals at risk, minimizing false negatives and facilitating the early detection of chronic conditions such as diabetes. This supports healthcare professionals by providing a reliable, reproducible, and clinically useful mechanism that favors timely preventive interventions. Likewise, having sensitive screening promotes self-care and helps encourage the adoption of healthy lifestyles among patients, aligning with international recommendations that highlight the importance of early detection tools to improve health outcomes and reduce the burden of disease [3].

Limitations

One of the main limitations of this study is the dependence on a dataset obtained from a single primary healthcare center. Although the database provides valuable clinical and sociodemographic information, relying on data from only one medical institution may limit the generalizability of the findings. In addition, part of the information included in the dataset was collected through patient surveys, which may introduce potential biases such as self-reporting inaccuracies or incomplete responses. Therefore, future studies should consider incorporating multicenter datasets and alternative sources of clinical data in order to improve the external validity and robustness of the predictive models.

The dataset used in this study was originally collected through convenience sampling from patients who visited a primary care clinic of the IMSS in Saltillo, Coahuila, Mexico. Although this approach facilitated access to real-world clinical data, it also introduces limitations related to external validity and the generalizability of the results. The study population may not fully represent the general population due to factors such as access to health services, institutional affiliation, frequency of visits, and regional sociodemographic characteristics.

Furthermore, the observed class imbalance reflects the clinical distribution of patients attending the healthcare facility rather than the actual prevalence of diabetes in the general population. To address this issue, the SMOTE was applied exclusively to the training set after the training/test split, minimizing data leakage and improving class balance during model training.

Despite these limitations, the use of real-world clinical data represents a significant strength, as it allows for the evaluation of predictive models under conditions closer to clinical practice.

Another limitation is the inherent trade-off between sensitivity and specificity observed. Strategies aimed at improving sensitivity, such as class balancing techniques and ensemble methods, resulted in a corresponding reduction in specificity. This behavior is common in predictive models for medical screening, where increasing the detection of individuals at risk may also increase the number of false positives. Although prioritizing sensitivity can be advantageous for early detection of diabetes risk, the reduction in specificity may lead to unnecessary follow-up evaluations. Consequently, future work should explore approaches that achieve a more balanced trade-off between these metrics to optimize the clinical applicability of the predictive models.

## Conclusions

The results of this study demonstrate that ML techniques have significant potential to predict diabetes risk. However, their performance is strongly affected by class imbalance. Although the evaluated models showed high effectiveness in identifying individuals without risk (class 0), achieving high values of precision, sensitivity, and F1-Score, their ability to detect individuals at risk (class 1) was notably limited, as reflected by low sensitivity values. This highlights the inadequacy of relying solely on global metrics such as accuracy in medical contexts and emphasizes the need to prioritize more informative measures such as sensitivity and specificity. Hybrid approaches, particularly model combination methods, showed promise in improving the detection of patients at risk, although in some cases at the cost of reduced specificity. Overall, these findings suggest that further optimization is required to improve the detection of the minority class and that the integration of advanced ML techniques within a multidisciplinary and ethically responsible framework can improve predictive performance, support clinical decision-making, and promote more effective evidence-based healthcare practices. The predictive performance obtained in this study should be interpreted with caution due to the observed trade-off between sensitivity and specificity. The proposed models should not be considered definitive diagnostic systems, but rather tools to support diabetes risk stratification and clinical decision-making. The model demonstrated a moderate ability to distinguish individuals at risk of diabetes, suggesting that further improvements are needed before potential clinical implementation. Furthermore, the exploratory nature of this study and the use of convenience sampling may limit the generalizability of the results to populations with similar clinical and sociodemographic characteristics.
